# Spatio-temporal changes in endosymbiont diversity and composition in the African cassava whitefly, *Bemisia tabaci* SSA1

**DOI:** 10.3389/fmicb.2022.986226

**Published:** 2022-11-18

**Authors:** Hajar El Hamss, M. N. Maruthi, Hadija M. Ally, Christopher A. Omongo, Hua-Ling Wang, Sharon van Brunschot, John Colvin, Hélène Delatte

**Affiliations:** ^1^Natural Resources Institute, University of Greenwich, Central Avenue, Chatham Maritime, Kent, United Kingdom; ^2^CIRAD, UMR PVBMT, Saint Pierre, France; ^3^Tanzania Agricultural Research Institute Ukiriguru, Mwanza, Tanzania; ^4^Root Crops Programme, National Crops Resource Research Institute (RCP-NaCRRI), Kampala, Uganda; ^5^The Ministry of Agriculture Key Laboratory of Molecular Biology of Crop Pathogens and Insects, Institute of Insect Sciences, Zhejiang University, Hangzhou, China; ^6^School of Biological Sciences, The University of Queensland, Brisbane, QLD, Australia

**Keywords:** cassava, Illumina HiSeq, temporal change, endosymbionts, whitefly

## Abstract

Sap-sucking insects, including whiteflies, are amongst the most devastating and widely distributed organisms on the planet. They are often highly invasive and endosymbiont communities within these insects help them adapt to new or changing environments. *Bemisia tabaci* (Gennadius; Hemiptera: Aleyrodidae) whitefly species are vectors of more than 500 known plant-viruses and harbour highly diverse endosymbionts communities. To date, however, whitefly–endosymbiont interactions, community structure and their spatio-temporal changes are still poorly understood. In this study, we investigated the spatio-temporal changes in the composition and diversity of bacterial endosymbionts in the agricultural crop pest whitefly species, *Bemisia tabaci* sub-Saharan Africa 1-subgroup 1 and 2 (SSA1-SG1 and SSA1-SG2). 16S rRNA amplicon sequencing analysis was carried out to characterise endosymbiont compositionsin field-collected SSA1 (SSA1-SG1 and SSA1-SG2) populations infesting cassava in Uganda in 1997 and 2017. We detected *Portiera*, *Arsenophonus*, *Wolbachia*, *Hamiltonella* and *Hemipteriphilus*, with *Arsenophonus* and *Wolbachia* infections being predominant. *Hemipteriphilus* and *Hamiltonella* frequencies were very low and were detected in seven and two samples, respectively. Bacterial diversity based on three independent parameters including Simpson index, number of haplotypes and Bray–Curtis dissimilarity matrix was significantly higher in 1997 than in 2017. This period also coincided with the advent of super-abundant cassava-whitefly populations on cassava crops in Uganda. We discuss how endosymbionts may influence the biology and behaviour of whiteflies leading to population explosions.

## Introduction

Whiteflies belong to the Sternorrhyncha suborder, which also includes aphids, psyllids, and mealybugs, all of which feed on plant phloem-sap (Gullan and Martin, 2009). Plant sap is generally lacking amino acid elements required for balanced insect nutrition (Douglas, 2006). As a result, all of these insects have a symbiotic relationship with bacteria called “endosymbionts.” Bacterial endosymbionts are generally localised in vesicles within specialised insect cells (bacteriocytes; Baumann, 2005; Douglas, 2016). In these cells, endosymbionts aggregate into a bacteriome within the body cavity to help them synthesise missing dietary elements and create a balanced diet (Buchner, 1965; Baumann et al., 2006; Douglas, 2016). Early light microscopy studies revealed that each of these insect groups has a morphologically identical endosymbiont (referred to as the primary endosymbiont [P-endosymbiont]) that is found in all members of the group (Buchner, 1965). Despite morphological similarity, primary (and secondary) endosymbionts are comprised of multiple species (across host taxa; Bell-Roberts et al., 2019). *Portiera aleyrodidarum*, for instance, is the primary endosymbiont of *Bemisia tabaci* (Santos-Garcia et al., 2012) whilst *Buchnera aphidicola* is the primary endosymbiont of aphids (Munson et al., 1991). Some members may contain secondary endosymbionts (S-endosymbionts), which are morphologically distinct from the primary endosymbionts (Buchner, 1965; Baumann et al., 2006; Luan et al., 2015). Both endosymbiont types are maternally transmitted to the next generations (Luan et al., 2018).

The whitefly *B. tabaci* is a highly invasive pest of economically important vegetable and ornamental crops worldwide (Liu et al., 2009). It causes serious damage to crops such as cassava by direct feeding and vectoring plant viruses that cause economically important diseases (Barro et al., 2011; Fiallo-Olivé et al., 2019). *B. tabaci* is a complex of at least 44 morphologically indistinguishable biological species that are distributed in the tropical and subtropical parts of the world and the protected environments of temperate regions (Tay et al., 2017). Amongst these, *B. tabaci* sub-Saharan Africa 1 (SSA1) is highly prevalent in sub-Saharan Africa. Based on the partial mitochondrial cytochrome oxidase I gene (mtCOI) marker, this species was divided into five subgroups (Ghosh et al., 2015). However, SSA1-SG3 has recently been considered a separate species based on mating incompatibility studies (Mugerwa et al., 2021) and molecular genetic analysis using specific microsatellite markers (Ally et al., 2019; Mugerwa et al., 2020, 2021).

Endosymbiotic bacteria are also widespread in whiteflies (Gueguen et al., 2010). They are present in the whitefly body cavity, haemolymph or intracellularly in special cells called bacteriocytes (Raina et al., 2015). *Portiera aleyrodidarum*, a P-endosymbiont present in all whiteflies, supplements the amino acid-deficient diets (Thao and Baumann, 2004). The S-endosymbionts such as *Rickettsia*, *Hamiltonella*, *Wolbachia*, *Arsenophonus*, *Cardinium,* and *Fritschea* are not present in all whiteflies but have a wide variety of roles such as affecting fitness, reproduction (Thierry et al., 2011), sex determination (Wang et al., 2020), insecticide susceptibility or virus transmission capabilities (Ghanim and Kontsedalov, 2009; Himler et al., 2011; Barman et al., 2021).

The infection dynamics of S-endosymbionts in *B. tabaci* SSA1 showed a high abundance of *Arsenophonus* and *Wolbachia* in Uganda (Legg et al., 2014; Ghosh et al., 2015). Ghosh et al. (2015) also found that about 62% of *B. tabaci* SSA1-SG1 individuals were infected with *Wolbachia*, *Arsenophonus* and/or *Rickettsia* in single or mixed infections in Uganda. Tajebe et al. (2015), however, found *Arsenophonus* as a single infection or coinfected with *Cardinium* in Tanzania, indicating the huge differences in the results obtained between the studies. In Nigeria, *Arsenophonus*, *Rickettsia*, *Wolbachia, Cardinium* and *Hamiltonella* were detected in *B. tabaci* SSA1 (Akintola et al., 2020). *Arsenophonus*, *Wolbachia*, *Rickettsia*, and *Cardinium* have been detected in *B. tabaci* SSA1 from Uganda and Tanzania on cassava (Sseruwagi et al., 2018). In addition, infection of *Rickettsia* increased in *B. tabaci* MEAM1 in the United States from 2000 to 2016, indicating a change in the interaction between S-endosymbiont and their host (Bockoven et al., 2019). *Hamiltonella* infection frequencies were significantly higher in MEAM1 and MED females compared to males, suggesting that the sex of the hosts could also influence S-endosymbionts infections (Pan et al., 2012). In this study, we investigated how the spatio-temporal changes have influenced the endosymbiont composition and diversity in the African cassava whitefly species, *B. tabaci* SSA1, using samples collected two decades apart (1997 and 2017) and from various different locations in Uganda.

## Materials and methods

### Whitefly sampling

**Figure 1:**
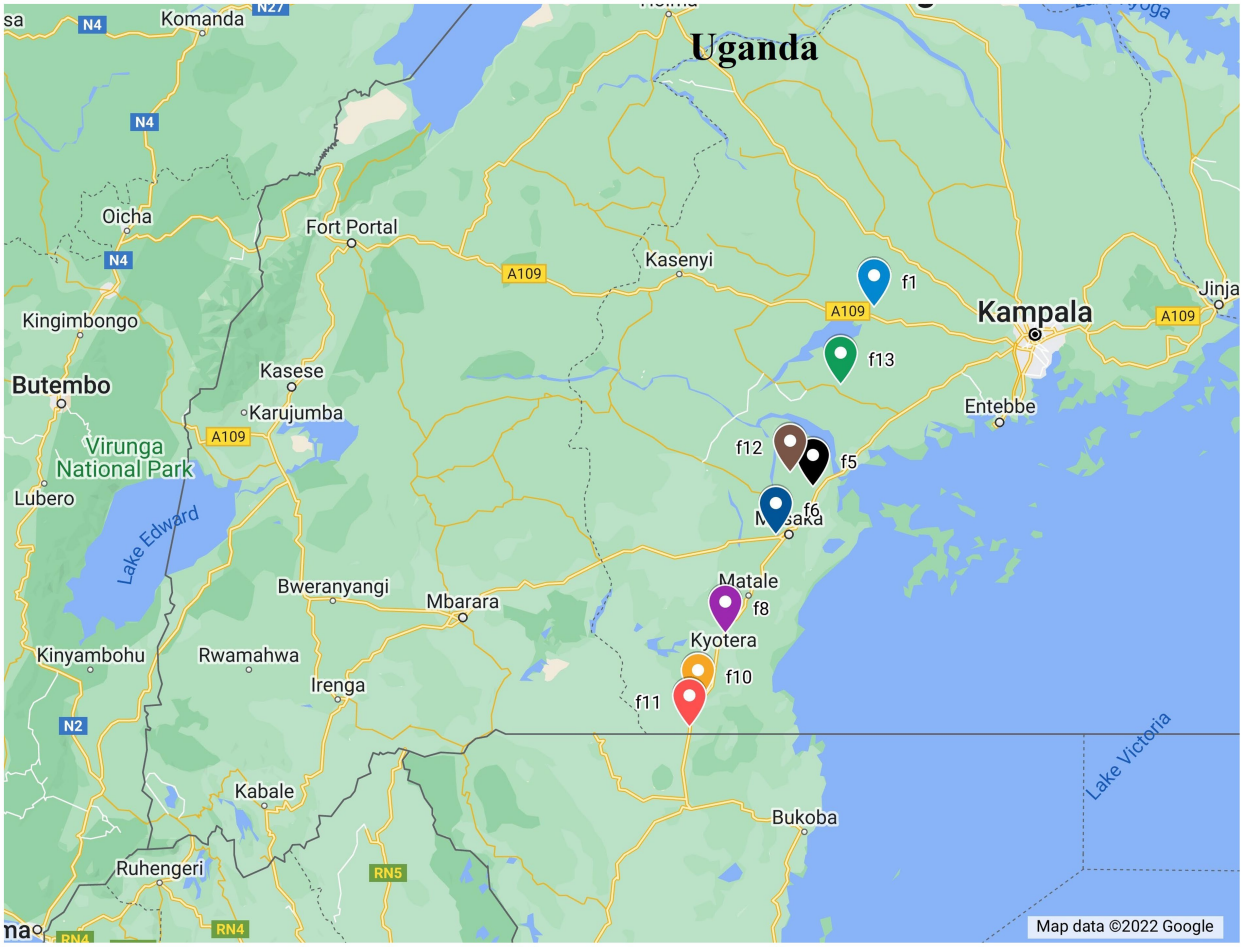
Map showing the distribution of whitefly sampling sites used in this study.

A total of 65 young cassava leaves having eggs, nymphs and pupae were collected from Uganda in 1997, and stored at −80°C at NRI, University of Greenwich, United Kingdom. Samples were collected in 14 locations (~ 5 Km apart) in three districts of Mityana, Kampala and Masaka. Another sampling was carried out in 2017 in 13 locations in a slightly larger area of seven districts (Mityana, Mpigi, Wakisa, Kalungu, Masaka, Rakai, and Gomba). A total of 48 *B. tabaci* adults on cassava were collected and preserved in ethanol for subsequent molecular analysis. GPS coordinates collected in 2017 were matched with the names of villages in the 1997 samples using QGIS v.2.18.17 online software^1^ (Ally et al., 2019; Figure 1; Supplementary Table S1).

### DNA extraction and molecular typing of *Bemisia tabaci*

For this study, the DNA was extracted from 121 whiteflies (Supplementary Table S1). Of these, 65 DNA from single whitefly were extracted from 1997 samples from individual eggs and nymphs of whiteflies using the Chelex method (Walsh et al., 1991; Ghosh et al., 2015). Briefly, each whitefly was ground in a 100 μl TE solution (10 mM Tris–HCl and 1 mM EDTA, pH 8.0) containing 20% Chelex (BIO-RAD, United Kingdom) and 300 μg Proteinase K. Samples were incubated at 58°C for 1.5 h followed by protein denaturation at 96°C for 10 min (Ghosh et al., 2015). Samples were then centrifuged at 13,000 rpm and the supernatant was collected and stored at −20°C. A total of 56 DNA samples collected in 2017 were obtained from a non-destructive method by overnight incubation of whiteflies in buffer solution, as previously described (Delatte et al., 2011; Ally et al., 2019). A total of 121 SSA1 samples (Supplementary Table S1) were selected from the two locations. Their identities were confirmed from their partial mtCOI sequences (Ally et al., 2019). DNA isolated from insects were amplified using the PCR genus-specific primer pair: 2195Bt (5′-TGRTTTTTTGGTCATCCRGAAGT-3′) and C012/Bt-sh2 (5′-TTTACTGCACTTTCTGCC-3′; Mugerwa et al., 2018). Amplification of *mtCOI* gene was carried out in 25 μl volumes consisting of 2 μl of DNA template, 0.4 μM of each primer, 0.15 mM of dNTPs, 1 × DreamTaq Green buffer and 0.5-unit DreamTaq Green DNA polymerase (Thermo Scientific Ltd., Uniteed Kingdom). The PCR amplification began with a denaturation step at 94°C for 3 min, followed by an amplification consisting of 38 cycles at 94°C for 30 s, an annealing step for 54 s, 72°C for 1.5 min and a final extension for 7 min at 72°C. PCR products were visualised on 1% agarose gels containing RedSafe nucleic acid staining solution (Intron Biotechnology, Korea). PCR products were then visualised under UV light (402 nm) and fragments of the expected size (~ 867 bp) were purified and sent for sequencing (Macrogen, Netherlands).

### 16S rRNA library preparation

The Hi-Seq Illumina platform with 466 bp paired-end reads was used for sequencing the V4–V5 region of the 16S rRNA gene as previously described (El Hamss et al., 2021). A duplicate PCR strategy was adopted involving two PCR amplifications per sample to decrease PCR selection bias. In every single round of PCR, primers containing the index sequences were used to prepare the 16S rDNA sequencing libraries. Each reaction contained 2 μl of template DNA, 1 U of Dream Taq DNA polymerase, 1 mM dNTPs, and 0.2 μM of each primer in a 25 μl reaction mixture. The PCR conditions started with an initial denaturation step at 95°C for 2 min, followed by an amplification of 38 cycles at 94°C for 30 s, an annealing step for 54 s, 72°C for 1.5 min and a final extension for 7 min at 72°C in a DNA Engine thermocycler (Applied Biosystems, United Kingdom). The dual-index paired-end sequencing approach was adopted for sequencing the 16S rRNA products (Caporaso et al., 2011, 2012). Both the reverse and forward primers consisted of eight nucleotides as index sequences, 4–5 nucleotides as a linker, followed by the gene-specific primer. The index sequences and linkers together formed the barcodes. The barcodes were unique to each sample. Following the separation of products from primers and primer dimers by 1% agarose gel electrophoresis, PCR products of the correct size 400 bp were recovered using an electrophoresis Gel and PCR purification kit (NucleoSpin, Macherey-Nagel, Germany). Samples were pooled into two separate pools (121 amplicons per pool) by mixing duplicate whitefly PCR products with unique indices in equal quantities. Total DNA was quantified on a qPCR machine (Biorad, CFX manager, United Kingdom) using PicoGreen dsDNA Quantification kit (Thermo Fischer Scientific, United Kingdom). A composite pooled sample, having 121 amplified DNA with positive control *Echerichia coli* to control bias from multiple 16S rRNA gene copies or other sequencing errors was then prepared. Single amplicons were combined in equimolar ratios was purified using the same Gel and PCR purification kit. Quantification of pooled DNA was performed using NanoDrop 2000 (Thermo Scientific, United Kingdom). The pooled samples were sequenced by Fasteris Ltd., Switzerland by Illumina sequencing.

### Sequence analysis

Paired-end reads were demultiplexed according to their barcode/primers using the software Metafast_BCsorting version (2.10; Ulyantsev et al., 2016), and the barcode sequences were subsequently removed from the output reads. Standard Illumina adapters and low-quality bases were trimmed using the Trimmomatic version 0.32 (Bolger et al., 2014). The Trimmomatic package was used to remove bases that were below the quality threshold and those that corresponded to the standard Illumina adapters. Trimmomatic parameters were selected so no mismatches were allowed in the barcode sequences, whereas in the primer sequences, the number of mismatches allowed was the number of degenerated bases + 2 mismatches.

Sequences were subjected to a second filtering process using the DADA2 plugin (Callahan et al., 2016) which was implemented in QIIME2 software v2018.6 (Bolyen et al., 2018). DADA2 enables additional read quality filtering and trimming, chimera filtering, denoising, and joining paired reads using the “Denoise and dereplicate paired-end sequences” method of QIIME2. Parameters of forward and reverse read truncation or length filtering were set to 0 while the other parameters were kept as defaut.

DADA2 generated Amplicon Sequence Variants (ASVs) and reported their relative abundance within each sample. VSEARCH consensus taxonomy classifier (Rognes et al., 2016) was used to assign taxonomy to ASVs using the SILVA database. The generated taxonomic groups of sequences were re-analysed to look for unique haplotypes using DNAsp software v6.12.0.3. Unique haplotypes were then generated with a matrix of read abundance using the gplot library in R (Warnes et al., 2009). The tree with unique haplotypes was constructed using maximum-likelihood method in Geneious tree builder, bootstrap was the resampling method and the number of replicates was set at 1,000,000 while other parameters were kept as default.

### Statistical analysis

Given the high prevalence of *Portiera*, it was excluded from the analysis to avoid skewing the results. Therefore, only S-endosymbionts were used in this analysis. Then, Vegan Library of R (Oksanen et al., 2014) and Capscale function (Oksanen et al., 2013) were used to investigate the effect of time and diversity indices including alpha diversity measured by Simpson, the number of haplotypes and Beta diversity measured by Bray–Curtis matrix was calculated on binary matrix of ASV. The non-metric Multi-Dimensional Scaling analysis (NMDS) was used to condense and therefore simplify multidimensional data about the samples into a few important axes to facilitate visualisation and interpretation. As microbiome dataset is compositional by nature with an irrelevant total number of counts within a sample, the centred log-ratio (CLR) transformation on ASV matrix of total reads was also applied to correct for biases (Greenacre et al., 2021). Aitchison distance between transformed samples was subsequently calculated and visualised using Principal Coordinate Analysis (PCoA).

One-way analysis of variance (ANOVA) was used to investigate mean differences between Simpson index and the number of observed haplotypes based on the 2 years of collection.

Analysis of Compositions of Microbiomes with Bias Correction (ANCOM-BC), a robust methodology of differential abundance for microbial absolute abundances, was used to further confirm the date effect on S-endosymbionts composition (Gloor et al., 2017). Permutational Multivariate Analysis of Variance (ADONIS) was used to analyse S-endosymbionts communities with environmental variables (date, space and life stage). Relative abundance of each haplotype in single whitefly sample was also calculated and the ASV matrix of abundance was transformed into a binary matrix before calculating Bray–Curtis matrix.

## Results

All individuals in this study were identified as *B. tabaci* SSA1-SG1 and SSA1-SG2 which are the same species (Mugerwa et al., 2021). Moreover, all of the whitefly individuals sampled in 2017 were adults, while whiteflies sampled in 1997 were at various juvenile life stages. It was, in fact, not possible to standardise the life stages across the 2 years included in the study. Nevertheless, S-endosymbionts are maternally transmitted, and therefore, we are not expecting them to be influenced by life stage. The results of the statistical tests also confirm this hypothesis as the life stage was not influencing the S-endosymbionts at both alpha diversity level (Shannon index: *p* = 0.2, Haplotype: *p* = 0.19) and beta diversity level (ADONIS: *p* = 0.06). The results presented here are, therefore, solely from both the temporal and spatial changes.

### The overall diversity of endosymbionts according to the time of the whitefly collection

**Figure 2:**
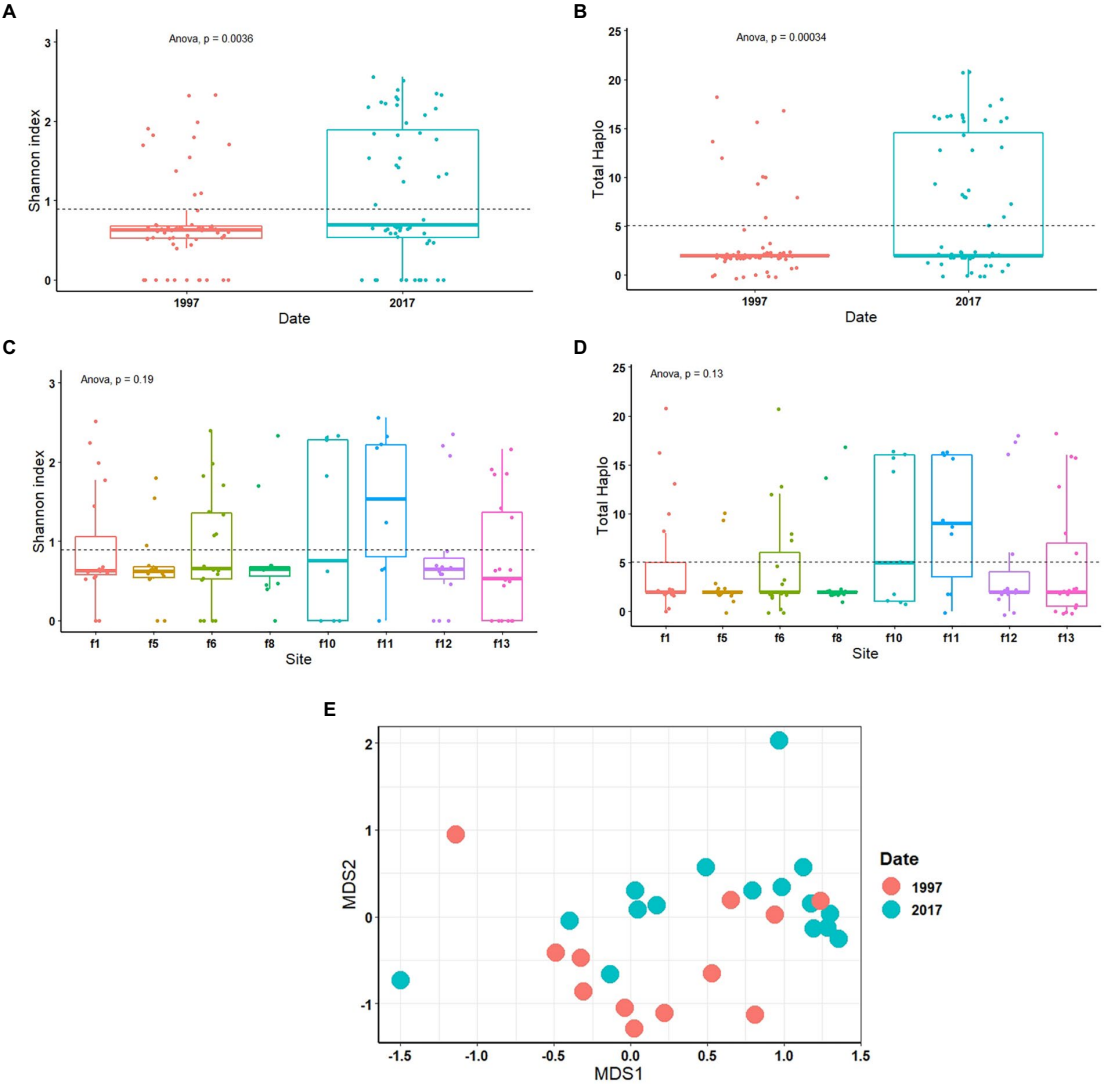
One-way ANOVA of diversity analysis including Shannon index and total haplotype in relation to date **(A,B)** and site **(C,D)**. Non-metric multi-dimensional scaling analysis (NMDS) test on Bray–Curtis matrix **(E)** of S-endosymbionts only. *Portiera* was excluded from the statistical tests.

The diversity of S-endosymbionts changed over time significantly both based on the Shannon index, *p* = 0.0036 (Figure 2A) and total haplotypes, *p* = 0.00034 (Figure 2B). Shannon index average value changed from 0.71 in 1997 to 1.09 in 2017 (Figure 2A). The S-endosymbiont diversity and composition did not change across sites based on Shannon index (*p* = 0.19; Figure 2C), total haplotypes (*p* = 0.13; Figure 2D) and Bray–Curtis matrix (*p* = 0.28). Haplotypes average changed from 3 in 1997 to 7 in 2017, while the Bray-Curtis matrix measuring S-endosymbiont structure was significantly different (*p* = 0.001; Figure 2E). Based on the NMDS, old samples, represented in red dots, are clustered meaning that they have similar compositions (Figure 2E). Furthermore, results of the PCoA on CLR-transformed data showed significant differences in S-endosymbionts between whiteflies collected in 1997 and 2017 (Supplementary Figure S1). These results showed that S-endosymbiont diversity in the *B. tabaci* SSA1 population had changed over time.

### The temporal prevalence of endosymbionts and their haplotypes

**Figure 3:**
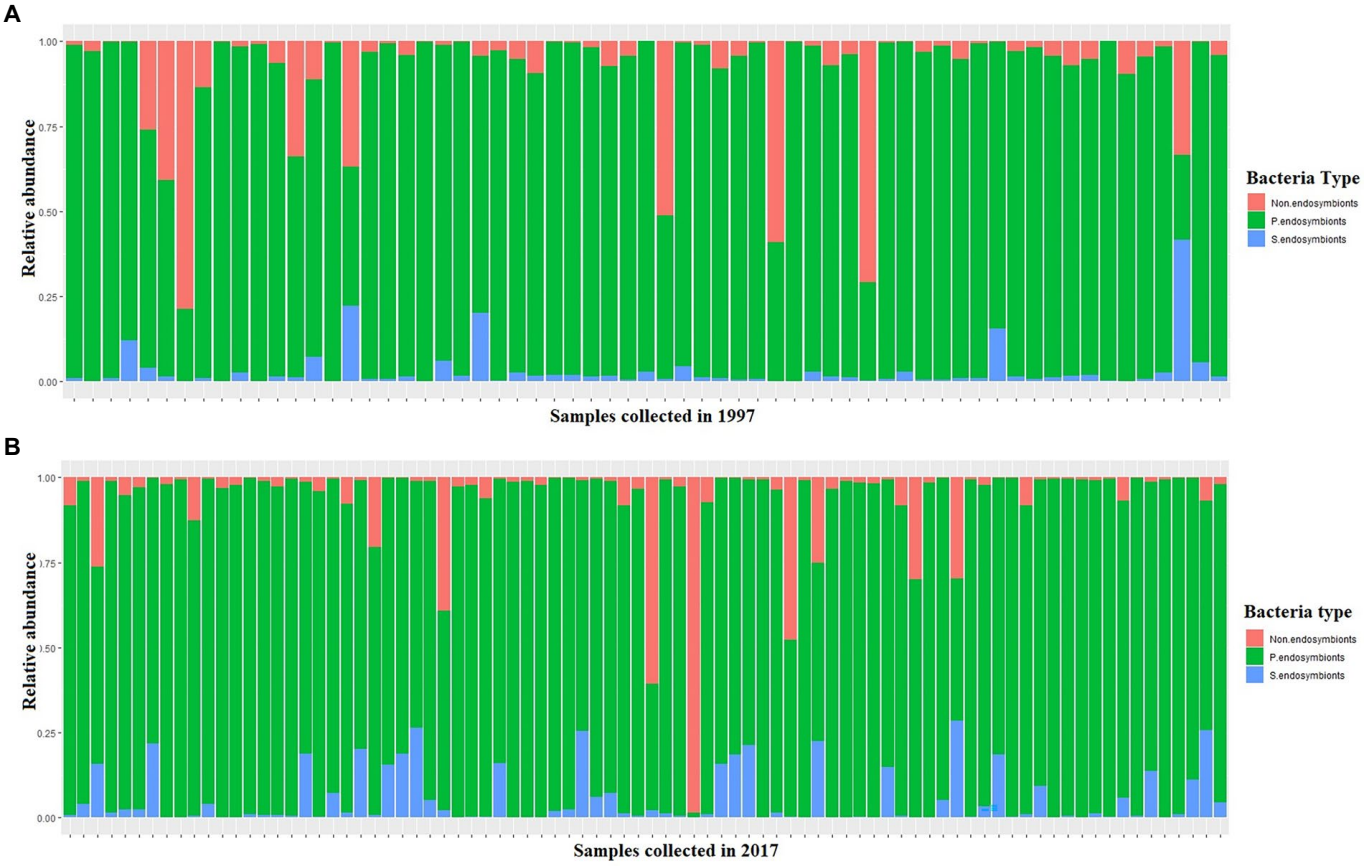
A stacked barplot of the three bacteria types that were detected in this study in each single whitefly showing their relative abundance in relation to date in 1997 **(A)** and 2017 **(B)**.

Total reads of 102,493,996 were generated from the 1997 (60,318,306) and 2017 (42,175,690) samples (Supplementary Table S1). Of the 102,493,996 total recovered reads after quality filtering, 96% (92,355,604) were assigned to *Portiera* whilst the remaining 4% (10,138,3,924) reads were assigned to S-endosymbionts and non-endosymbiotic bacteria (Figure 3). Amongst *Portiera* sequences, 19 haplotypes were found in this study, with three of them; Por-Hap_3, and Por-Hap_1 detected in one sample collected in 1997, and Por-Hap_2 detected in three SSA1 whiteflies, but with low frequencies (Figure 3), whereas the others with high frequency were prevalent on both dates of collection (Figure 2; Supplementary Table S1). Their accession numbers were deposited in the GENEBANK database (accession numbers from OP160987 to OP160997).

**Figure 4:**
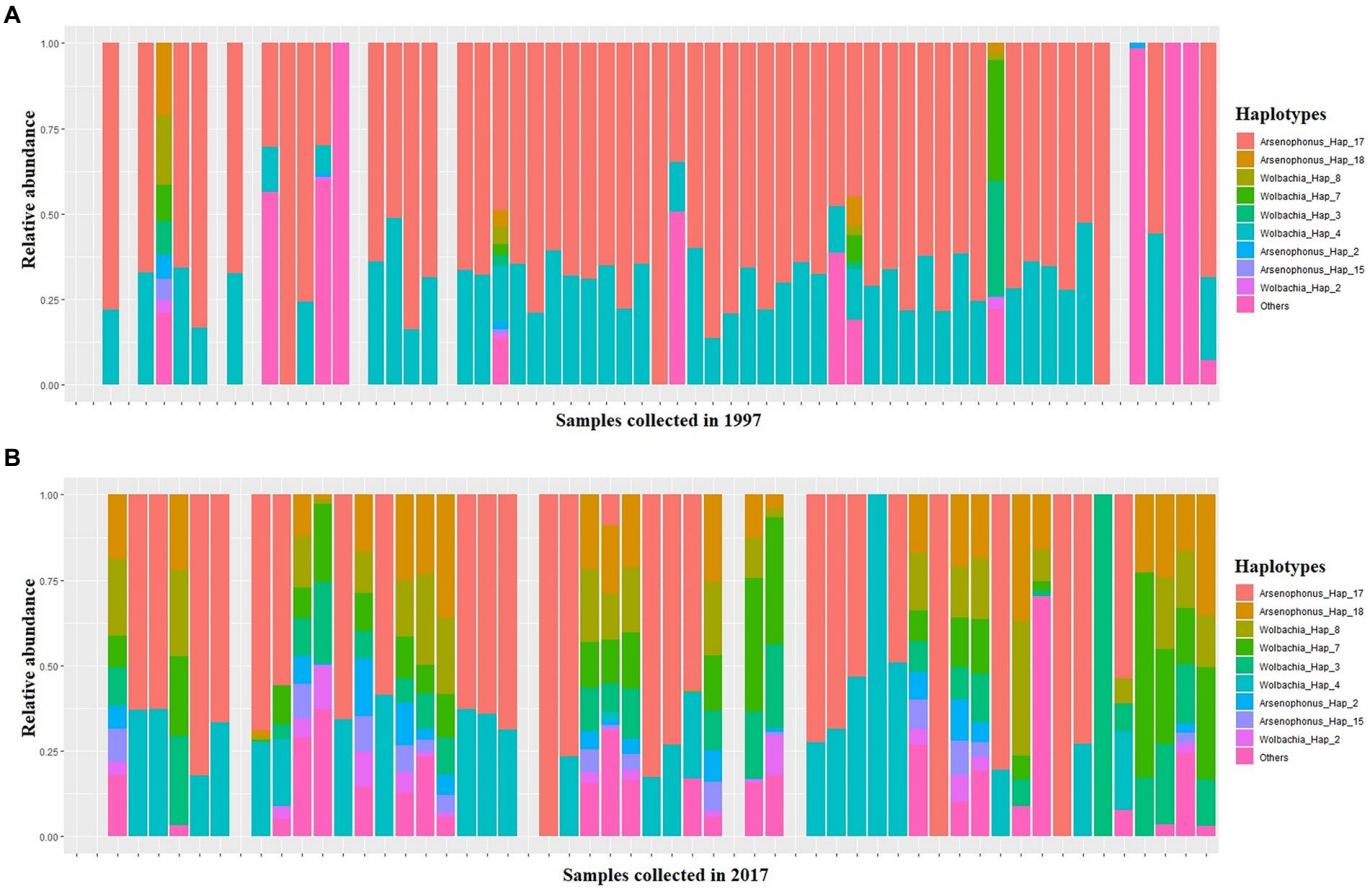
A stacked barplot of S-endosymbiont haplotypes detected in this study in each single whitefly which exhibit significantly different relative abundance in relation to date, as indicated by ANCOM-BC (Supplementary Table S5), in 1997 **(A)** and 2017 **(B)**.

Four S-endosymbionts including *Arsenophonus Wolbachia*, *Hemipteriphilus* and *Hamiltonella* were detected in our samples with different prevalences (Figure 4). The analysis of bacterial composition with bias correction (ANCOM-BC) showed that the relative abundances of 23 haplotypes belonging to *Arsenophonus* and *Wolbachia* changed significantly between 1997 and 2017 in tested SSA whiteflies (Figure 4; Supplementary Table S2).

A total of 69 samples harboured 100% of the relative abundance of *Arsenophonus* (OP160971 to OP160986), 49 samples in 1997 and 20 in 2017 (Figure 4). In both years, *Arsenophonus* was the most abundant S-endosymbiont followed by *Wolbachia* (OP161006–OP161013) which was observed in 5 whitefly samples with relative abundance ranging between 25 to 91% in 1997 and 28 samples in 2017 having a relative abundance ranging from 0.2 to 100% (Figure 4). *Hemipteriphilus* (OP160998 to OP161005) was least abundant in both years present in four samples in 1997 with relative frequency ranging between 3 and 47% and only present in one sample from 2017 with relative frequency reaching 7% (Figures 4). *Hamiltonella* (OP160970) was present in one whitefly sample only from the 2017 collections (Figure 4).

*Arsenophonus* sequences showed the highest haplotype diversity, assigned to 18 haplotypes. Amongst them, only two were found in 1997 collections with both occurring in three samples (Figure 4). The top abundant haplotype was Ars_Hap_7 as the relative frequency assigned to this haplotype was between 0 and 100% in the 1997 samples and 0–100% in the 2017 samples (Figure 4).

*Wolbachia* and *Hemipteriphilus* sequences were less diverse, as eight haplotypes were detected for each bacteria (Supplementary Table S1). Five *Hemipteriphilus* haplotypes were found only in 1997 but not in 2017. Hemi-Hap_39, Hemi-Hap_42 (Hemi-Hap_43 Hemi-Hap_44, Hemi-Hap_45) occurred in less than three samples. *Wolbachia* prevalence was different between dates but the diversity did not change over time (Figure 2; Supplementary Table S1). Two haplotypes, Ham-Hap_46 (25) and Ham-Hap_47 belonging to *Hamiltonella* sequences were found only in two samples in 2017, but not in 1997 (Supplementary Table S1). All the sequences generated were deposited in the Genebank database with their accession numbers.

### Infection dynamics of S-endosymbionts and their phylogeny at haplotype level

The infection dynamics between the three endosymbionts *Arsenophonus*, *Wolbachia* and *Hemipteriphilus* changed over time (Table 1). Dual infections of *Wolbachia* and *Arsenophonus* changed from 9 to 28% from 1997 to 2017 (Table 1). *Wolbachia* was not found in 1997 (0%) but represented 5% of reads in 2017 (Table 1). Single infection of *Arsenophonus* changed from 91 to 68% from 1997 to 2017 (Table 1). S-endosymbiont-free whiteflies were found in both years with 12 and 17% in 1997 and 2017, respectively (Table 1).

**Table 1:** Temporal change of S-endosymbiont coinfections.

Infection status	S-endosymbionts collected from Whiteflies in 1997 (%)	S-endosymbionts collected from Whiteflies in 2017 (%)
*Arsenophonus*	91	68
*Wolbachia*	0	5
*Arsenophonus* and *Wolbachia Wolbachia*	9	28
*N*	12	17

*N*: no infection showing symbiont free whiteflies.

**Figure 5:**
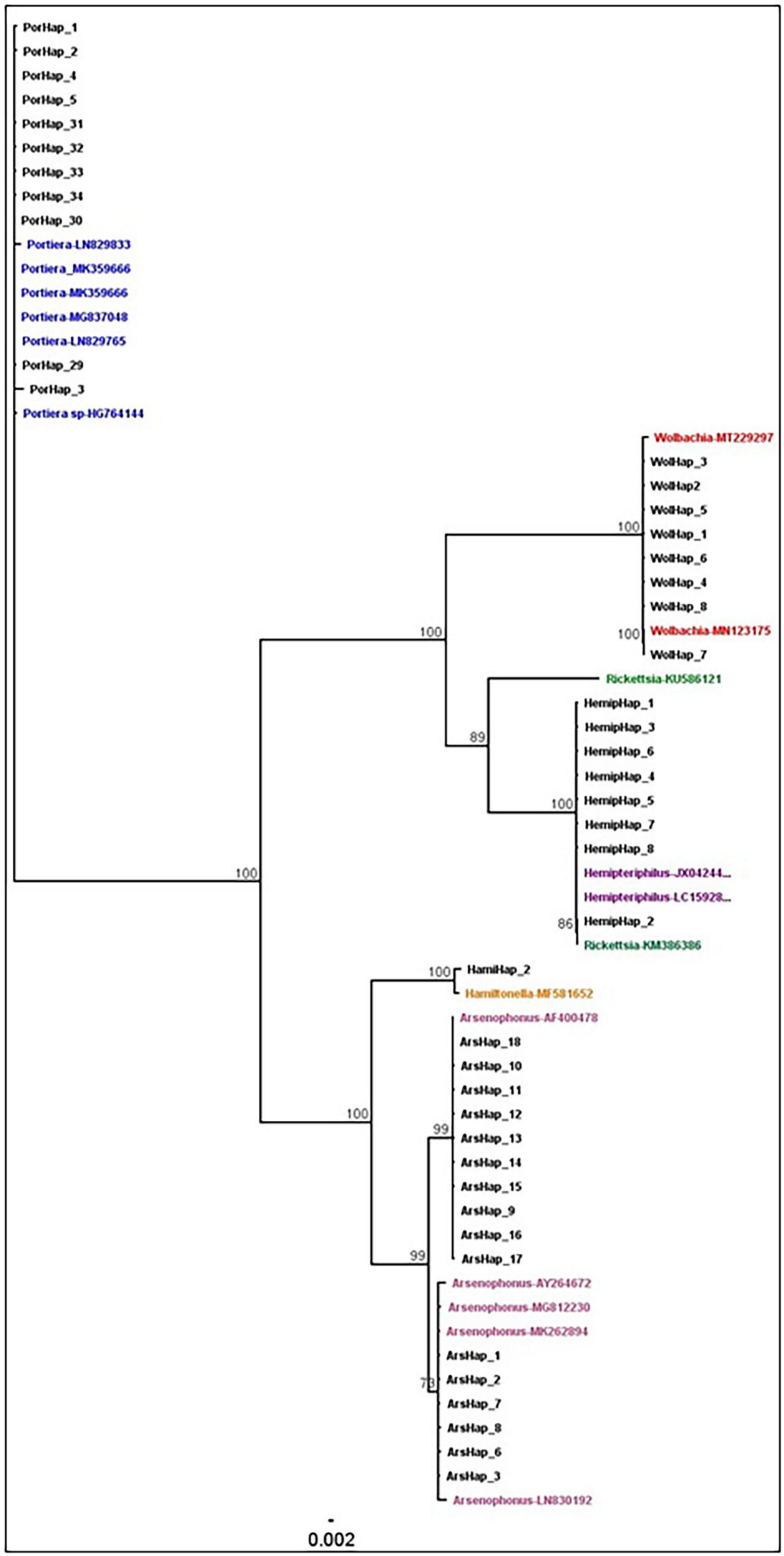
Phylogenetic analysis of the endosymbionts haplotypes based on the region V4–V5 in 16S rRNA sequences using the neighbour-joining method with 1,000,000 bootstraps in Geneious. The coloured names showed the reference sequences with their accession numbers all the black names were generated from this study.

**Figure 6:**
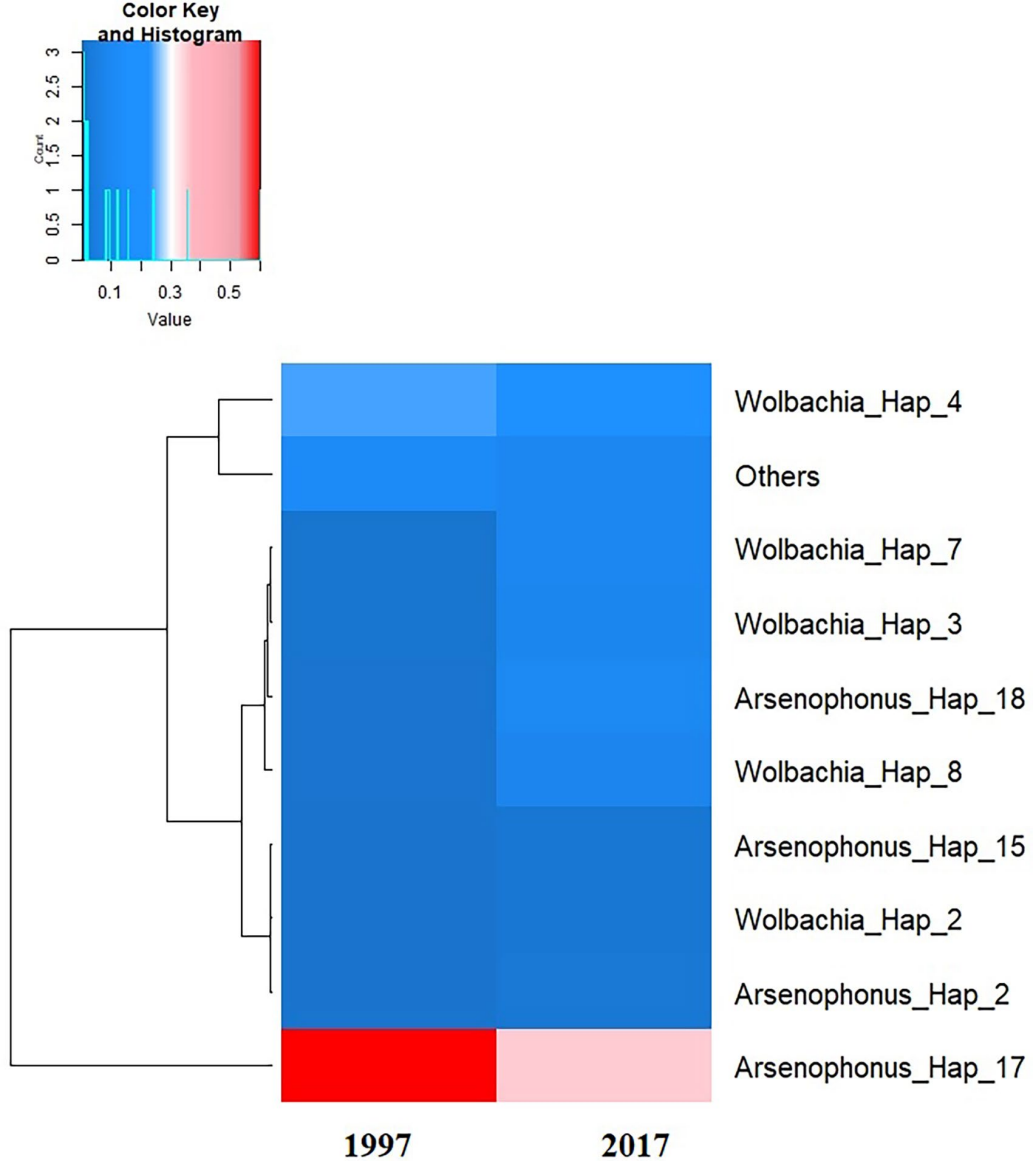
Heat map of the average of the relative abundance of S-endosymbiont haplotypes detected according to the date of the investigations. The dendrogram on the left was constructed based on the overall relative frequency of each haplotype, meaning that the haplotypes which are clustered together have similar overall relative prevalence.

The phylogenetic relationships between and within S-endosymbionts showed that, apart from *Hemipteriphilus*, each S-endosymbiont clustered separately and, as expected, with their given reference sequences (downloaded from the GENBANK; Figure 5). Two clusters of *Arsenophonus* were present in the tree, suggesting the high genetic diversity of this symbiont (Figure 3). *Hemipteriphilus* was clustered with one *Rickettsia* sequence, suggesting the need to further investigate other genes of *Hemipteriphilus*. A heat map of the relative abundance showed the temporal variations of all detected bacteria at the haplotype level (Figure 6).

## Discussion

Despite their importance to whitefly, relatively little is known about the endosymbionts in African cassava whitefly populations. We present here, for the first time, the full endosymbiont diversity and composition in *B. tabaci* SSA1 by deep sequencing of 16S rRNA gene, using samples collected at different time points in Uganda. We found that the S-endosymbiont diversity in the *B. tabaci* SSA1 population has changed since 1997 and that this has happened in parallel with the increased whitefly populations attacking cassava crops.

Before discussing these findings, it is important to note that reads produced by Illumina sequencing create large amounts of data with errors that are difficult to differentiate from real biological variation. To overcome this challenge, DADA2 filtering was adopted to clean Illumina errors following the parametric error model (err). Reads with number of expected errors higher than 2 were discarded. Nevertheless, other errors or misinterpretations can still persist, notably, 16S rRNA gene copies that can occur in some bacteria species, such as polyploidy (Sun et al., 2013; Větrovský and Baldrian, 2013; Espejo and Plaza, 2018; Louca et al., 2018). In this study, we further used *Echerichia coli* as the mock community in all pools of our samples to further confirm the assignment of reads.

This work is also the first study to investigate intraspecies diversity of *Portiera* within *B. tabaci* SSA1. We discovered six prevalent *Portiera* haplotypes whose relative frequency was stable between the two dates of investigation. *Portiera* 16S rRNA was also found to contain a highly homologous sequence (Paredes-Montero et al. (2020)). However, we found intraspecies diversity in their variable regions V4-V5 within the 16S rRNA gene sharing 99% identity, which is similar to the 0–0.67% divergence reported by Paredes-Montero et al. (2020), suggesting *Portiera* evolution is ongoing (Santos-Garcia et al., 2020). The divergence between *Portiera* variants is concordant with the long period of coevolution between the host and endosymbiont.

The differences found between the study in Paredes-Montero et al., 2020 and our studies could be due to the different methodologies adopted and the depth of sequences obtained. We used amplicon sequence variants (ASV) showing the differences of the V4-V5 region in 16S rRNA in *Portiera* sequences and obtained deeper sequencing than any previous studies. The differences in diversity metrics of S-endosymbionts observed between Ghosh et al. (2015) and Tajebe et al. (2015) in seemingly similar whitefly populations from East African cassava whitefly populations, highlighted the sensitivity of these types of analyses to the methodology used in detecting endosymbionts. Nevertheless, our approach detected all previously known endosymbionts associated with whiteflies. A standardised set of protocols is therefore needed to detect and identify reliably the various endosymbionts infecting insect species. This conclusion is further emphasised by Ghosh et al. (2015), reporting three S-endosymbionts (*Wolbachia*, *Arsenophonus* and *Rickettsia*) in *B. tabaci* SSA1 using a simple PCR and sequencing approach, while we found a new and different combination of S-endosymbionts (*Arsenophonus Wolbachia, Hemipteriphilus and Hamiltonella*). *Hemipteriphilus* was found for the first time in seven samples and *Hamiltonella* in a single sample in African whiteflies. This is the first study to detect *Hemipteriphilus* in African whiteflies. *Fritschea* and *Cardinium*, however, were not detected in this study. Previous studies did not find *Hemipteriphilus* in *B. tabaci* SSA1, whilst *Rickettsia* infection was less than 1% (Ghosh et al., 2015), further highlighting high endosymbiont diversity in African cassava whiteflies and differences in the methodologies used.

The high depth of sequences obtained here also allowed us to investigate intraspecies diversity within each S-endosymbiont. This is to show that if there are multiple genetic variants within the population, we might predict that the presence/absence and relative abundance of different haplotypes would show variation among whitefly samples harbouring a particular species of the endosymbiont. Similarly, three strains of *Hemipteriphilus* were detected in one study on MED Q1 and MED Q3 and ASL whiteflies using three genes including 16S rRNA gene (483 bp), GroEL (269 bp) and GltA (190 bp; Mouton et al., 2022).

The two most prevalent *Arsenophonus* haplotypes (amongst 18) were present on both tested dates. While the two least prevalent *Arsenophonus* haplotypes were present only in 1997, they were not detected in 2017 specimens. Three *Wolbachia* haplotypes were highly prevalent among the eight detected, but they did not change between the two dates. In this study, three *Hemipteriphilus* haplotypes were also found in only 1997. Little is known about the intraspecies diversity of *Hemipteriphilus* in African whiteflies, which was first characterised as *Candidatus Hamiltonella defensa* (Bing et al., 2013) in China 1 whiteflies and subsequently found only in Asian *B. tabaci*. In that study using both 16S rRNA and gltA genes suggested that *Hemipteriphilus* is clustered within the Alphaproteobacteria subdivision of Proteobacteria. Here, we found that *Hemipteriphilus* sequences were clustered with one strain of *Rickettsia.* Another study in Burkina Faso also identified *Hemipteriphilus* similar to *Rickettsia* using 16S rRNA gene in Illumina sequencing technology in MED-Q1, MED-Q3 and ASL genetic groups (Mouton et al., 2022). The sequences of *Hemipteriphilus* and *Rickettsia* are so similar that they are difficult to differentiate especially using 16S rRNA primers. This issue could be due to the specificity of the primers used to detect *Hemipteriphilus*. When a mismatch within V4-V5 sequences occurs, it reduces the thermal stability of the primer-template duplex, thus affecting PCR specificity. Nevertheless in this study, some *Hemipteriphilus* sequences were not differentiated from *Rickettsia*, the use of other housekeeping genes (Christensen et al., 2004; Farré et al., 2007) is therefore needed to further study the inter- and intra-species diversity of *Hemipteriphilus* in African whiteflies. These observed similarities and differences between *Hemipteriphilus* and *Rickettsia* imply that the group is not well defined and probably in need of a taxonomic update.

In this study, we found that S-endosymbionts diversity and composition were not significantly different across sites in SSA1 whiteflies. In previous work, it was reported that site-to-site variations subsequently influence the prevalence of endosymbionts in a given whitefly population (Zchori-Fein et al., 2014). Endosymbionts composition of over 2000 studied whiteflies derived from several independent screenings was positively correlated with the distance from the equator (Zchori-Fein et al., 2014). Similarly in China, *Wolbachia* and *Rickettsia* were influenced by geography and host plants (Pan et al., 2012). These differences between studies could be linked to the adopted sampling. In our work, the sampling was carried out in Uganda with sites only 5 km apart from each other, compared to other studies where samplings were done at the country level. *Rickettsia* was also shown to boost its invasive ability within *B. tabaci* by the increase of infection frequency from 1% in 2000 to 97% in 2006 in Arizona (Himler et al., 2011).

Our results demonstrate the dynamic nature of bacterial endosymbionts. Both single and dual infections of *Wolbachia* changed in SSA1 over time. Dual infection of *Arsenophonus* and *Wolbachia* also changed from 9 to 28%. In contrast, single infections of *Arsenophonus* changed from 91 to 68%. *Wolbachia* and *Arsenophonus* are reported to coexist in other insect species such as brown planthopper (*Nilaparvata lugens*; Guo et al., 2021) and ants (Wang et al., 2016). *Portiera* genome is highly reduced and lacks genes involved in the synthesis of certain vitamins, cofactors, and some essential amino-acids, suggesting that there is a metabolic niche that could be filled by secondary endosymbionts (Rao et al., 2015). Similarly, the co-occurrence of both *Arsenophonus* and *Wolbachia* has been found in different whitefly species from China MEAM1 and MED (Chu et al., 2011) and East Africa in SSA1-SG2, but their role is not well understood. At the metabolic pathway level, both *Arsenophonus* and *Wolbachia* influence whiteflies nutritionally by synthesising vitamin B (Santos-Garcia et al., 2018; Zhu et al., 2022). Their effect on *B. tabaci* SSA1 should further be investigated.

In this study, a single infection of *Wolbachia* changed from 0 to 5%. This change may be an indication of the emerging role of *Wolbachia* in *B. tabaci* SSA1 or the beginning of a new invasion by these *Wolbachia* species. *Wolbachia* is well known to influence the reproduction of their host by reproductive incompatibility within and between insect species including *B. tabaci* SSA1 and *B. tabaci* SSA1-SG2. The latter harboured *Wolbachia* (Mugerwa et al., 2020), suggesting that the incompatibility is species dependent. Nevertheless, the change in *Wolbachia* infections over time in field-collected whiteflies can have implications on their reproduction that should be investigated further.

A total of 12 and 17% of the samples were S-endosymbionts free in 1997 and 2017, respectively. Similarly, Ghosh et al. (2015) also showed about 38.0% of SSA1 was free from S-endosymbionts. Whiteflies without any S-endosymbionts in them can be found in both field and laboratory whiteflies (El Hamss et al., 2021). Some S-endosymbiont have a negative effect on whitefly development and their removal from whiteflies increased the number of eggs and nymphs laid as well as decreased adult emergence time (Ghosh et al., 2017). Generally, S-endosymbiont’s role is species-specific and therefore may or may not be present depending on the whitefly species. Spatio-temporal change of endosymbionts can be linked to their facultative or vital presence in whiteflies. In this study, S-endosymbionts diversity and composition in *B. tabaci* SSA1 individuals changed over time, coinciding with the advent of whitefly outbreaks in Uganda. This whitefly upsurge was estimated at more than 1,000 adults per top five leaves (Legg and Raya, 1998; Colvin et al., 2004; Legg et al., 2006). The reasons behind whitefly outbreaks have not been fully understood and one explanation for this high whitefly infestation was related to synergistic interactions between S-endosymbionts providing a high fitness advantage to their whitefly hosts. In one study, infection of *B. tabaci* MEAM1 by *Rickettsia* produced more eggs and females with higher survival to adulthood which led to its invasion across the southwestern United States (Himler et al., 2011). The presence of *Arsenophonus* or other hidden S-endosymbiont in *B. tabaci* SSA1 whiteflies may be responsible for improved fecundity and survival as suggested in previous studies (Ghosh et al., 2015; Tajebe et al., 2015). Both a high frequency of *Arsenophonus* and high haplotype diversity were detected from SSA1 in this study, suggesting that this S-endosymbiont can potentially affect whitely behaviour and abundance. In one reciprocal crossing experiment, *Arsenophonus,* the most prevalent S-endosymbiont in Ugandan *B. tabaci* SSA1, influenced whitefly reproduction (El Hamss et al., 2021).

Using a population dynamics technique, we looked at endosymbiont diversity and composition. We demonstrated how these discoveries are helping us get closer to our aim to better understand factors changing endosymbiont communities in whiteflies. This research, therefore, adds to our understanding of how S-endosymbiotic communities change over time. Further research is now needed to understand in more depth the role played by the diverse endosymbiont communities in African cassava whitefly outbreaks.

## Data availability statement

The data presented in the study are deposited and available in the GenBank repository, accession number from OP160970 to OP161013.

## Author contributions

JC, HD, and MM: conceptualisation. HH, CO, and HA: data collection. HH, H-LW, SB, HD, and MM: data analysis and interpretation. HH: writing—original draft. MM and HD: writing, review and editing. JC, MM, and CO: funding acquisition. All authors contributed to the article and approved the submitted version.

## Funding

This work was supported, in whole or in part, by the Bill & Melinda Gates Foundation [Grant Agreement OPP1058938]. Under the grant conditions of the Foundation, a Creative Commons Attribution 4.0 Generic License has already been assigned to the Author Accepted Manuscript version that might arise from this submission.

## Conflict of interest

The authors declare that the research was conducted in the absence of any commercial or financial relationships that could be construed as a potential conflict of interest.

## Publisher’s note

All claims expressed in this article are solely those of the authors and do not necessarily represent those of their affiliated organizations, or those of the publisher, the editors and the reviewers. Any product that may be evaluated in this article, or claim that may be made by its manufacturer, is not guaranteed or endorsed by the publisher.

## Footnotes

^1^ https://qgis.org

## Supplementary material

SUPPLEMENTARY FIGURE S1 Principal Coordinate Analysis (PCoA) calculated based on Aitchison community dissimilarity distance matrix of CLR-transformed 16s rRNA gene read data generated from Illumina of S-endosymbionts in single whitefly collected in two different time points (1997 and 2017).

SUPPLEMENTARY TABLE S1 Sample information, overall reads and Amplicon Sequence Variants (ASVs) table of reads of microbiome generated from each whitefly sample using16S rRNA amplicon sequencing.

SUPPLEMENTARY TABLE S2 ANCOM-BC test results for differential abundance of S-endosymbionts that differed significantly in abundance levels between dates.
