# Supplementary figures and images for: Spatio-temporal changes in endosymbiont diversity and composition in the African cassava whitefly, Bemisia tabaci SSA1
Source: Front Microbiol. 2022 Nov 18;13:986226. doi: 10.3389/fmicb.2022.986226 (PMC9715980; doi:10.3389/fmicb.2022.986226)

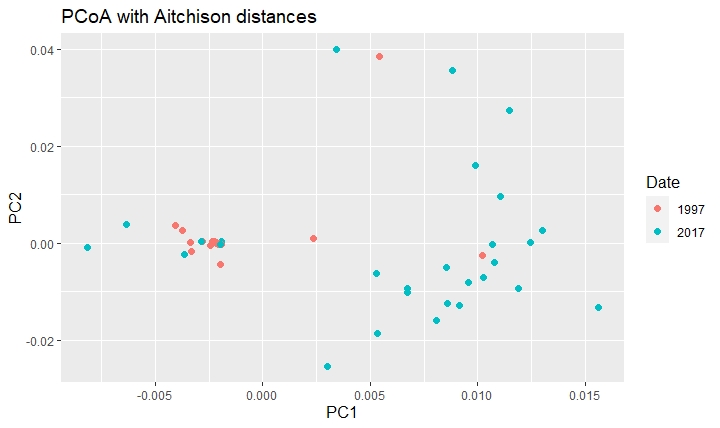

Supplement: SUPPLEMENTARY FIGURE S1 — Principal Coordinate Analysis (PCoA) calculated based on Aitchison community dissimilarity distance matrix of CLR-transformed 16s rRNA gene read data generated from Illumina of S-endosymbionts in single whitefly collected in two different time points (1997 and 2017). [file Image_1.JPEG]
